# The rectangular tile classification model based on Sentinel integrated images enhances grassland mapping accuracy: A case study in Ordos, China

**DOI:** 10.1371/journal.pone.0301444

**Published:** 2024-04-16

**Authors:** Fuchen Guo, Liangxin Fan, Weinan Chen, Dongyang Xiao, Haipeng Niu

**Affiliations:** 1 School of Surveying and Land Information Engineering, Henan Polytechnic University, Jiaozuo, Henan, China; 2 Research Centre of Arable Land Protection and Urban-Rural High-Quality Development of Yellow River Basin, Henan Polytechnic University, Jiaozuo, China; 3 School of Geological Engineering and Geomatics, Chang’an University, Xi’an, China; Van Lang University: Truong Dai hoc Van Lang, VIET NAM

## Abstract

Arid zone grassland is a crucial component of terrestrial ecosystems and plays a significant role in ecosystem protection and soil erosion prevention. However, accurately mapping grassland spatial information in arid zones presents a great challenge. The accuracy of remote sensing grassland mapping in arid zones is affected by spectral variability caused by the highly diverse landscapes. In this study, we explored the potential of a rectangular tile classification model, constructed using the random forest algorithm and integrated images from Sentinel-1A (synthetic aperture radar imagery) and Sentinel-2 (optical imagery), to enhance the accuracy of grassland mapping in the semiarid to arid regions of Ordos, China. Monthly Sentinel-1A median value images were synthesised, and four MODIS vegetation index mean value curves (NDVI, MSAVI, NDWI and NDBI) were used to determine the optimal synthesis time window for Sentinel-2 images. Seven experimental groups, including 14 experimental schemes based on the rectangular tile classification model and the traditional global classification model, were designed. By applying the rectangular tile classification model and Sentinel-integrated images, we successfully identified and extracted grasslands. The results showed the integration of vegetation index features and texture features improved the accuracy of grassland mapping. The overall accuracy of the Sentinel-integrated images from EXP7-2 was 88.23%, which was higher than the accuracy of the single sensor Sentinel-1A (53.52%) in EXP2-2 and Sentinel-2 (86.53%) in EXP5-2. In all seven experimental groups, the rectangular tile classification model was found to improve overall accuracy (OA) by 1.20% to 13.99% compared to the traditional global classification model. This paper presents novel perspectives and guidance for improving the accuracy of remote sensing mapping for land cover classification in arid zones with highly diverse landscapes. The study presents a flexible and scalable model within the Google Earth Engine framework, which can be readily customized and implemented in various geographical locations and time periods.

## Introduction

Arid zone grassland is one of the most vulnerable ecosystems on the planet, facing multiple challenges from global climate change and human activities. It plays an important role in food supply [1], soil conservation [2], water conservation and biodiversity conservation [3]. In addition, grassland resources have substantial cultural and economic value for local communities. Over the last few decades, overgrazing [4], agricultural expansion [5], urbanisation [6] and mining [7] have caused large changes in land cover (LC). An accurate LC classification map is important basic data for land managers and scientific researchers [8]. LC maps have been used extensively for research in land management, agricultural monitoring, ecosystem service assessment and climate change assessment [9]. Accurate, timely information on grassland area inventory is important for local government authorities, which provides a reliable basis for the rational development of grassland ecosystems and the macroscopic management of grassland resources.

Traditionally, grassland resource survey is archived by substantial field survey and measurement, which can accurately collect grassland spatial distribution and vegetation cover data. However, extensive fieldwork poses an immense challenge to human resources and is often labour intensive and time consuming [10]. With the rapid development of remote sensing technology and the free availability of satellite images, the scientific community has a unique opportunity to conduct mapping research in natural grasslands [11]. Currently, the available LC products have achieved remote sensing classifications on global [12, 13], national [14, 15] and regional [16, 17] scales at 30 m resolution. The producer accuracy of grassland ranges from 34.88% [12] to 88.01% [15] depending on the scale of study. The variation in producer accuracy for grassland is easily affected by the spatial heterogeneity of grassland [18]. Hou et al. [18] compared and analysed the consistency and accuracy of ten LC products, and concluded China urgently needs to develop a new grassland map. To validate the grassland accuracy of three existing land cover datasets (Chinese Academy of Sciences land use data (CNLUCC) [15] in 2018, Wuhan University annual land use data in China (WHCLCD) [14] in 2018, and GlobeLand30 [13] in 2020) in Ordos, 1,000 independent samples of visual interpretation were used to construct an error matrix, which revealed that the F1 scores for grassland ranged from 62.06% to 74.18%. However, it was observed that the F1 scores for grasslands were slightly low in the context of local scale study. Therefore, the utilization of high spatio-temporal resolution remote sensing satellite images for the refined extraction of grassland in arid and desertified regions is valuable and meaningful.

Optical remote sensing images are widely used in grassland mapping and monitoring [4]. Liu et al. [19] used Gaofen-1 satellite normalised difference vegetation index (NDVI) time-series data set with 8 m resolution to extract the spatial distribution information of the arid alfalfa artificial grassland in Linxi County, China, and the grassland patch accuracy reached 89.47%. However, the method has great limitations in natural grassland recognition and extraction due to the regular shape of the artificial grassland boundary and the fixed harvesting period. Herrero et al. [20] found that in discrete classification based on Landsat 8 OLI imagery with 30 m resolution, the support vector machine (SVM) algorithm and spectral features cannot be performed in southern African savanna land classification, and the classification accuracy was only 34.48%. The classification accuracy reached 79.31% after adding multitemporal NDVI and black decay temperature data to the classification features. The research of Herrero et al. [20] showed the increase in classification features’ dimensionality helps improve classification accuracy. The above studies were conducted for different types of grasslands, and the results showed remote sensing imagery with high spatial resolution can help improve mapping accuracy.

The quality of optical remote sensing images cannot be guaranteed because they are susceptible to the interference of clouds and rain [7, 21], which limits the remote sensing mapping of grassland. Compared with optical imagery, synthetic aperture radar (SAR) imagery has the advantage of all-day, all-weather acquisition and can be used as a supplementary data source to optical imagery. However, the signal of SAR image data is susceptible to interference from coherent speckle noise, which affects the identification of target features [22], and accurately extracting features from single-temporal-phase SAR data is difficult. Taravat et al. [23] explored the feasibility of the multitemporal SAR imagery of Sentinel-1 for the automatic detection of grass mowing status, with an overall accuracy of 85.71% for the validation set. The research result of Taravat et al. indicated the feasibility of using multitemporal SAR imagery for grassland growth state monitoring. Shafizadeh-Moghadam et al. [24]confirmed that the use of multi-temporal data improves the accuracy of land cover mapping. In the study of multi-temporal winter wheat mapping, Li et al. [25] utilised the NDVI curves of Modis images to segment the time window of synthetic images, and the results showed that the optimal selection of the time window helps to improve the accuracy of winter wheat mapping. The NDVI curve is useful for identifying the optimal time window for winter wheat mapping, but NDVI is susceptible to interference from soil background information in arid desert grasslands. To address this issue, four vegetation indices (NDVI, MSAVI, NDWI, and NDBI) were used to filter the time window and reduce interference from soil background information. Synthesising multi-temporal images using the optimal time window can help to address the issue of information loss that occurs when using a single synthesised image.

Optical remote sensing images and SAR images have their advantages, and their effective combination can improve the accuracy of grassland mapping, which has been successfully applied to grassland spatial distribution information extraction. Hong et al. [26] combined optical and SAR images to extract the spatial distribution of grassland and alfalfa in the southern prairie region of Saskatchewan, Canada, using unsupervised classification. In (SAR HV+MODIS) data combination mode, the grasslands’ user accuracy was highest at 89.7%, and that of alfalfa was 71.4%. In a single MODIS data model, the user accuracies of grasslands and alfalfa were 76.9% and 71.4%, respectively. Samrat et al. [27] extracted fragmented grassland patches in the tropics and found the overall accuracy was 96.32% for the combined-image classification, 91.93% for Sentinel-2 image and 63.23% for Sentinel-1 image. The above research results show the integrated images have different degrees of improvement in the accuracy of grassland mapping compared with single optical remote sensing images and SAR images. By contrast, de Oliveira Santos et al. [28] used seasonal multisensor image time series for the classification of crops, pastures and afforestation in subtropical agricultural regions, and found the addition of Sentinel-1 images does not improve the classification accuracy. Based on the literature review, the integration of optical images and SAR images is proposed as a solution to enhance the extraction accuracy of natural grasslands in arid areas, leveraging their respective advantages.

Currently, the choice of ML algorithms directly influences the grassland mapping accuracy. In a study comparing random forest (RF), SVM, decision trees (DTs), and artificial neural networks (ANNs) classifiers for Landsat-based LULC mapping, Shih et al. [29] discovered that RF had the highest average overall accuracy (OA) of 71.50%. Furthermore, RF required less computing time compared to the other classifiers. In Heydari’s study on mapping large areas of land cover using deep neural networks and Landsat time-series observations [30], it was observed that deep neural network algorithms do not enhance the accuracy of mapping solely through spectral feature classification when compared to traditional SVM algorithms. Furthermore, the study found that deep neural networks necessitate more intricate parameter settings. RF is known for robustness to noise and their ability to handle overfitting, thus having strong generalizability and transferability [31]. However, RF algorithm is easily affected by the unbalanced ratio between training sample classes. RF classifiers are constructed by minimising the overall classification error, and unbalanced training samples can lead to poor accuracy for a few classes [32]. The class imbalance problem occurs when one or some classes have fewer samples than others, and learning from unbalanced training data is a common problem in machine learning [8]. In Ordos, the proportion of grassland area reaches more than 50%, and the imbalance between grassland and other land cover types is unavoidable. To alleviate the problem of unbalanced training samples, scholars have explored techniques, such as undersampling of majority classes [33], oversampling of minority classes [34], and a combination of oversampling and undersampling training classes [35]. An incorrect sampling strategy affects the performance of the model, which increases training complexity and leads to overfitting problems [36]. Thus, the sampling strategy of unbalanced training samples must be investigated to improve the accuracy of LC types with small area proportions. To address unbalanced sampling during model training, Zhang et al. [37] proposed a random sampling strategy with the hexagonal tile model to improve LC mapping accuracy in Madagascar with highly heterogeneous landscapes. The producer accuracy of LC based on the traditional global classification model of the classifier was 84.8%, and the producer accuracy of LC based on the tile classification model was 88.2%. Inspired by the study of Zhang et al. [37], the rectangular tile classification model is proposed based on Sentinel integrated images in the study of grassland mapping in arid zones in anticipation of improving the accuracy of grassland mapping.

The Ordos Plateau is in the southwest of the Inner Mongolia Autonomous Region, which is a typical agro-pastoral zone and sensitive to climate change and human activities. From southeast to northwest, the grasslands of the Ordos Plateau are divided into typical grassland subzone, desert grassland subzone and grassland desertification subzone [38]. Its landscape features are highly heterogeneous and fragmented [39], which brings some difficulties to accurate grassland mapping in this region. To enhance the accuracy of Ordos grassland mapping and address the problem of unbalanced training samples, the rectangular tile classification model based on Sentinel integrated images is proposed in this paper. The research objectives of this paper are as follows: (1) validate the effectiveness of the rectangular tile classification model in improving the accuracy of grassland mapping in arid regions with highly heterogeneous landscapes, (2) determine whether the overall accuracy of grassland mapping in arid regions can be improved by combining Sentinel-1A and Sentinel-2 images and (3) determine the potential of vegetation index features and texture features in improving mapping accuracy.

## Study area and data collection

### Overview of the study area

**Fig 1:**
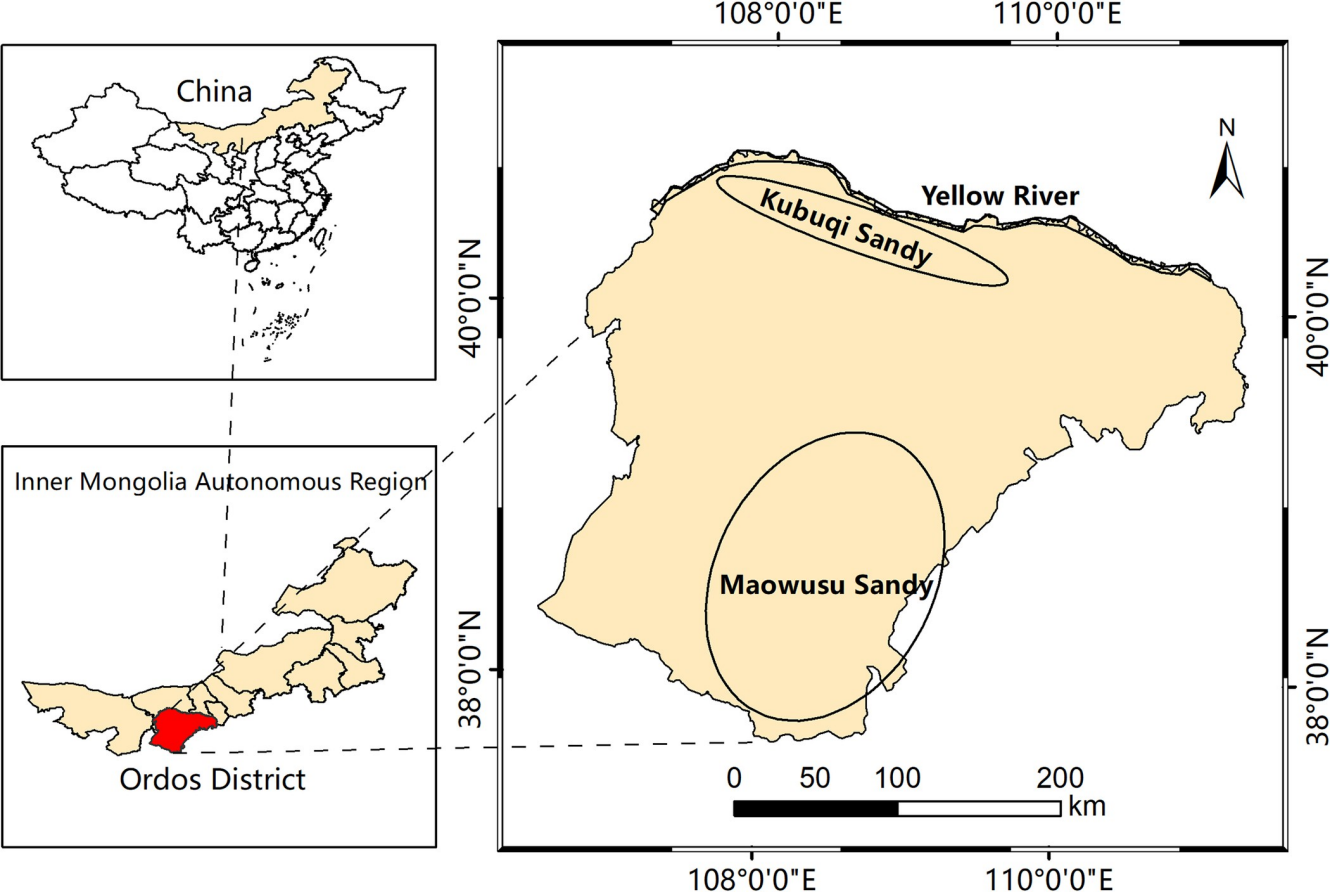
Study area.

The Ordos Plateau is in the southwest of Inner Mongolia, China. The regional latitude and longitude range are 106°42′ E–111°31′ E and 37°41′ N–40°51′ N, respectively (Fig 1). The study area is about 360 km long from north to south and 430 km wide from east to west, with a total area of 86,859 km^2^ and an average altitude of 1,000–1,500 m. By the end of 2021, the resident population of Ordos was 2.168 million people, the regional gross domestic product (GDP) was 68.61 billion USD and the GDP per capita was 31,639 USD [40]. The natural geographical environment of the Ordos Plateau is unique. The topography is high in the west and low in the east. The region has a temperate continental arid climate. The average precipitation in the east is 300–400 mm, and that in the west is 190–300 mm. The annual potential evaporation is as high as 2,000–3,000 mm. The annual average temperature is 5.3°C–8.7°C, the average wind speed is 2.7–3.7 m/s and the annual gale days are about 40 d. Kubuqi Sand in the north and Maowusu Sand in the southcentral are the two main geomorphic units in the study area, accounting for 48% of the total area, with sparse vegetation and a harsh natural environment. The alluvial plain along the Yellow River in the north, which accounts for 4% of the total land area, is fertile and rich in agricultural and livestock products. The eastern part is a hilly, gully area with serious soil erosion, and the ecological environment is fragile, accounting for 19% of the total land area. The western part is an undulating plateau area, accounting for 29% of the total land area. It is a typical desert grassland with minimal rainfall [41].

### Data and preprocessing

#### Sentinel data and preprocessing

The sentinel data used in this paper include Sentinel-1A and Sentinel-2, which are from the European Space Agency. The Sentinel-1A [42] active microwave remote sensing satellite carries a sensor of SAR in the C-band, which has a revisit cycle of 12 d. Ground range detected (GRD) products with a resolution of 10 m and polarisation of VV (vertical vertical) and VH (vertical horizontal) in Interferometric Wide Swath (IW) mode were selected as data sources. On the GEE platform, GRD products have completed preprocessing, such as radiation correction, thermal noise removal and terrain correction. Data loading can be performed by using only the relevant functions, which greatly reduces the difficulty of data processing. Sentinel-2 [43] consists of two high-resolution satellites, Sentinel-2A and Sentinel-2B, with a revisit period of 10 d for a single satellite. The two satellites complement each other, and the revisit cycle is reduced to 5 d. The two satellites are equipped with multispectral imagery (MSI) sensors with 13 bands, including visible, near-infrared (NIR) and short-wave infrared, with spatial resolutions of 10, 20 and 60 m. The reflectance product (MSI L1C image) with orthorectification and geometric correction was selected as the data source. The cloud amount of this image was less than 15%, and the QA60 band was used for cloud removal. A total of 934 Sentinel-2 MSI L1C images from May 9, 2018, to November 2, 2018, and 212 Sentinel-1A GRD images from May 1, 2018, to October 31, 2018, were used in this paper (Table 1). During data preprocessing, the 10 m resolution VV and VH bands of Sentinel-1A, and the 10 m resolution blue, green, red and NIR bands of the Sentinel-2 were resampled to 20 m.

**Table 1:** Satellite image data.

Sensors	Band	Resolution	Resampling		Image Acquisition Date	Number of Images
Sentinel-1A	VV	10 m	20 m		1 May-31 October, 2018	212
Setinel-2	Blue	10 m	20 m		9 May-2 November, 2018	934
	Green	10 m	20 m			
	Red	10 m	20 m			
	Near-infrared	10 m	20 m			
	Short-wave InfraredⅠ	20 m				
	Short-wave InfraredⅡ	20 m				
MODIS	Blue	500 m			6 March-27 December, 2018	38
	Green	500 m				
	Red	500 m				
	Near-infrared	500 m				
	Short-wave InfraredⅠ	500 m				
	Short-wave InfraredⅡ	500 m				

#### MODIS data

MODIS images acquired by Terra satellite, which had a spatial resolution of 500 m and a temporal resolution of 8 d, were used in this paper [44]. The MODIS time-series image data were used to calculate the vegetation indices, and the optimal synthetic time window for Sentinel-2 images was determined by time-series vegetation index curves. The S–G filtering model [45] was used to smooth the anomalous noise of the time-series vegetation indices to obtain vegetation index curves that are more consistent with the grassland phenology. This method eliminates the influence of cloud cover and atmospheric factors on the time-series vegetation index data and facilitates the determination of more accurate image synthesis time windows. Thirty-eight MODIS images from March 6, 2018 to December 27, 2018, were used in this paper (Table 1).

#### Sample data

**Fig 2:**
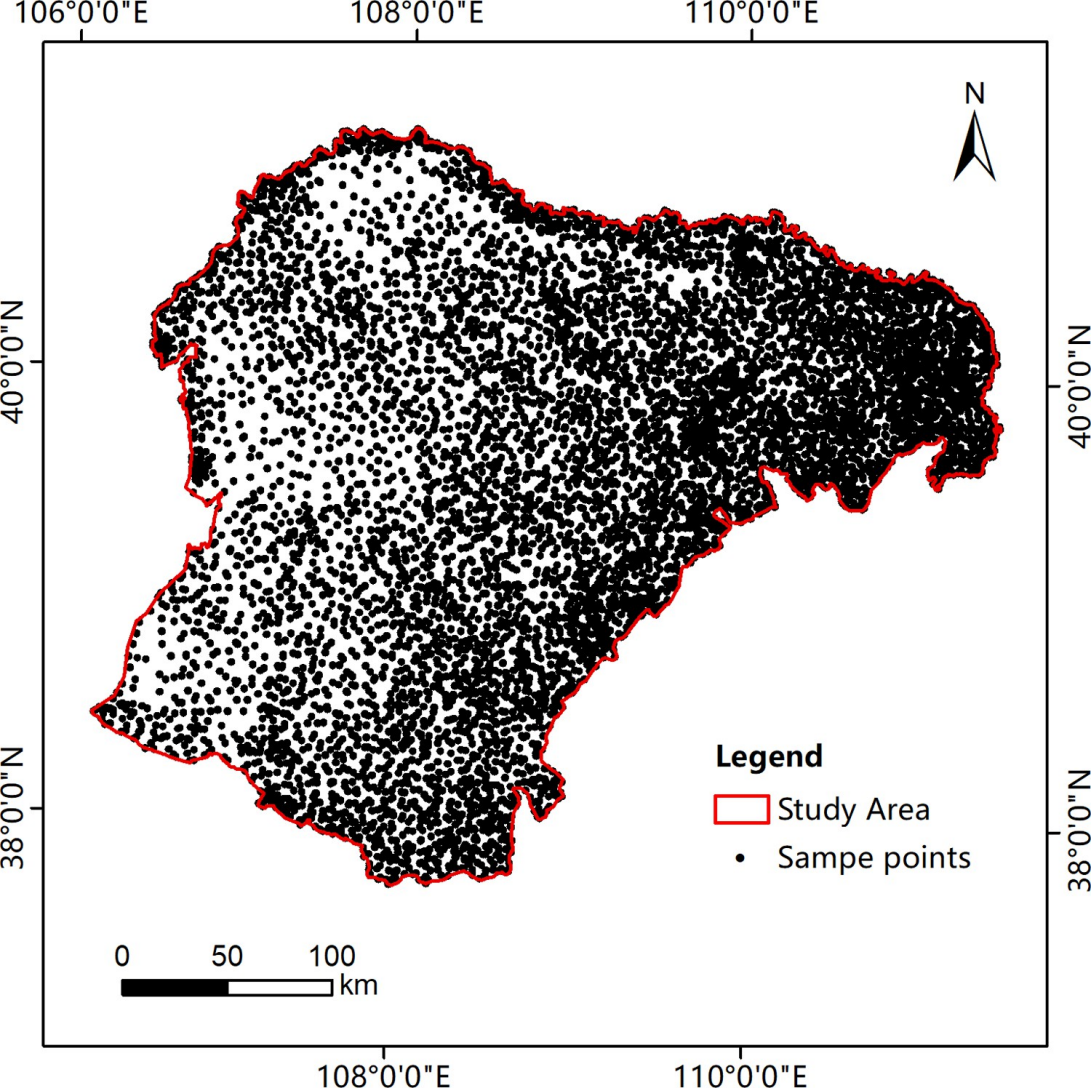
The spatial distribution of all sample points.

Existing Chinese LC products were used for analysis to determine the major LC distribution in the study area, and sample data were randomly generated based on the results. LC types were classified into six major categories: grassland, cultivated land, woodland, waterbody, building and unused land. The 2018 Chinese Academy of Sciences land use data (CNLUCC) [15] and Wuhan University annual land use data in China (WHCLCD) [14] were reclassified based on LC types, and their spatially identical parts were manually clicked to generate 7,938 sample points, ensuring the uniformity of the spatial distribution of sample points. The sample types and numbers are shown in Table 2. The spatial distribution of all sample points is shown in Fig 2. The sample points were corrected by visual interpretation based on the GEE platform. The sample data were imported into the GEE platform. Approximately 70% and 30% of the sample data were used as training set for model construction and validation set for classification accuracy verification, respectively.

**Table 2:** Sample points of the study.

Types of land cover	Description	Number of sampled points
Cultivated land	Land for cultivating crops	1798
Woodland	Tree woodlands, shrublands	1048
Grassland	Herbaceous vegetation, including pasture-dominated scrub grassland and open forest grassland	2750
Waterbody	Rivers, reservoirs, lakes, and ponds.	517
Building	Urban land, rural settlements, and other construction lands	525
Unused land	Sparse vegetation, dominated by a background of bare soil, sand, and gravel	1300

## Methodology

**Fig 3:**
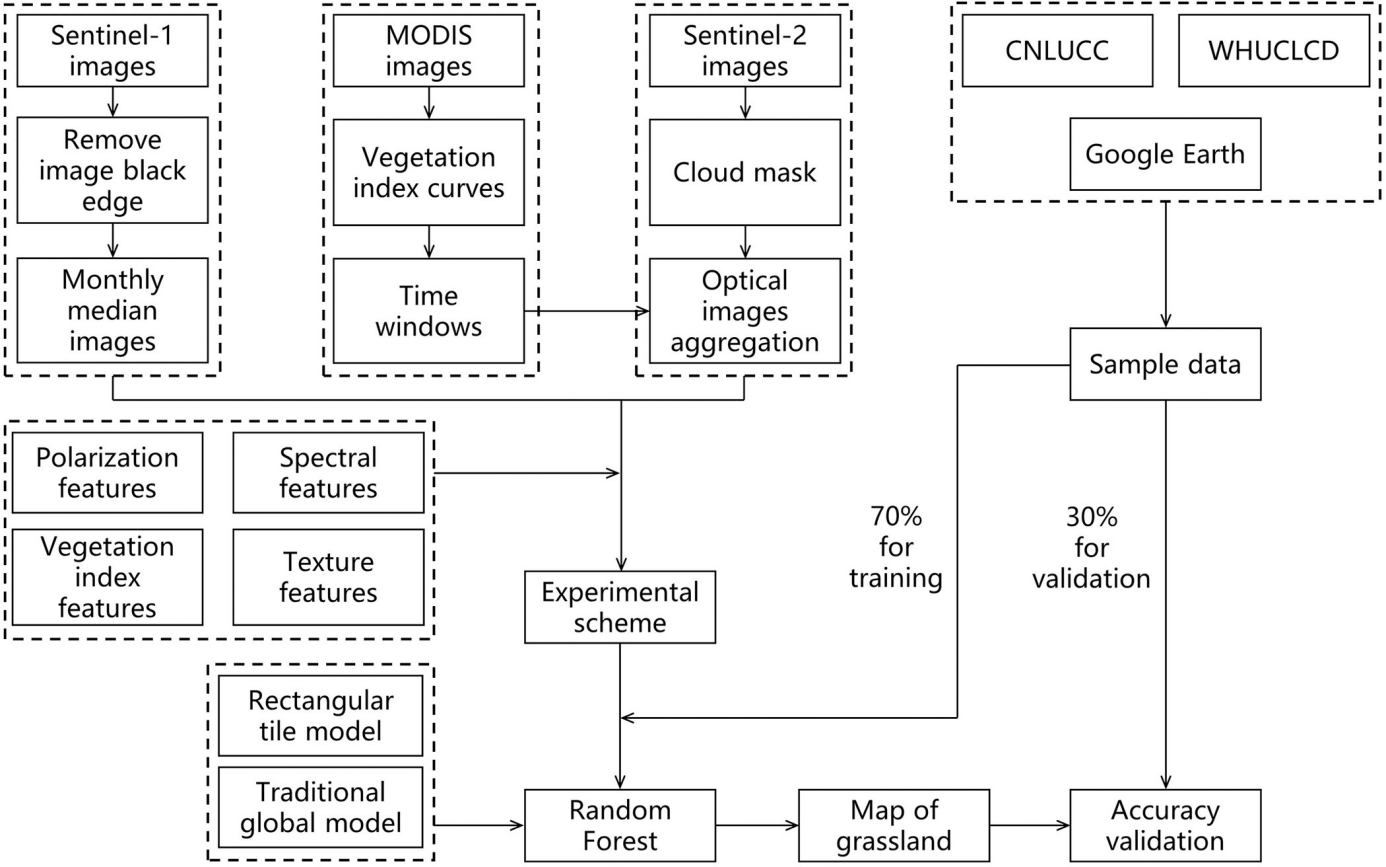
Flow chart of the data process. Note: Abbreviation: CNLUCC, Chinese Academy of Sciences land use data; WHUCLCD, Wuhan University annual land use data in China. The rectangular tile model represents the study area divided into rectangles of equal size, and the traditional global model represents the study area not divided into tiles.

In this paper, the classification task was employed on the GEE platform (https://earthengine.google.com/). The basic flow of the research is shown in Fig 3. The monthly Sentinel-1A median value images were synthesised, and the optimal synthesis time window of Sentinel-2 images was determined by MODIS NDVI, modified soil adjusted vegetation index (MSAVI), normalised difference water index (NDWI) and normalised difference built-up index (NDBI) of mean value time-series curves for grassland (Methodology: Image Composition Solution). The polarisation features, spectral features, vegetation index features and texture features were constructed as classification feature sets (Methodology: Selection of Feature Variables). Seven experimental groups (Methodology: Experimental Scheme) were designed, including the rectangular tile classification model and the traditional global classification model. The spatial distribution map of Ordos grassland was drawn based on the rectangular tile classification model (Methodology: Rectangular Tile Classification Model) and the RF algorithm (Methodology: RF Algorithm), and its classification accuracy was evaluated (Methodology: Accuracy Evaluation).

### Image composition solution

Different image synthesis schemes were proposed for SAR images and optical images, considering the characteristics of different types of sensors. The key time period of grass growth (May to October 2018) was selected to synthesise monthly Sentinel-1A median value images for six periods.

**Fig 4:**
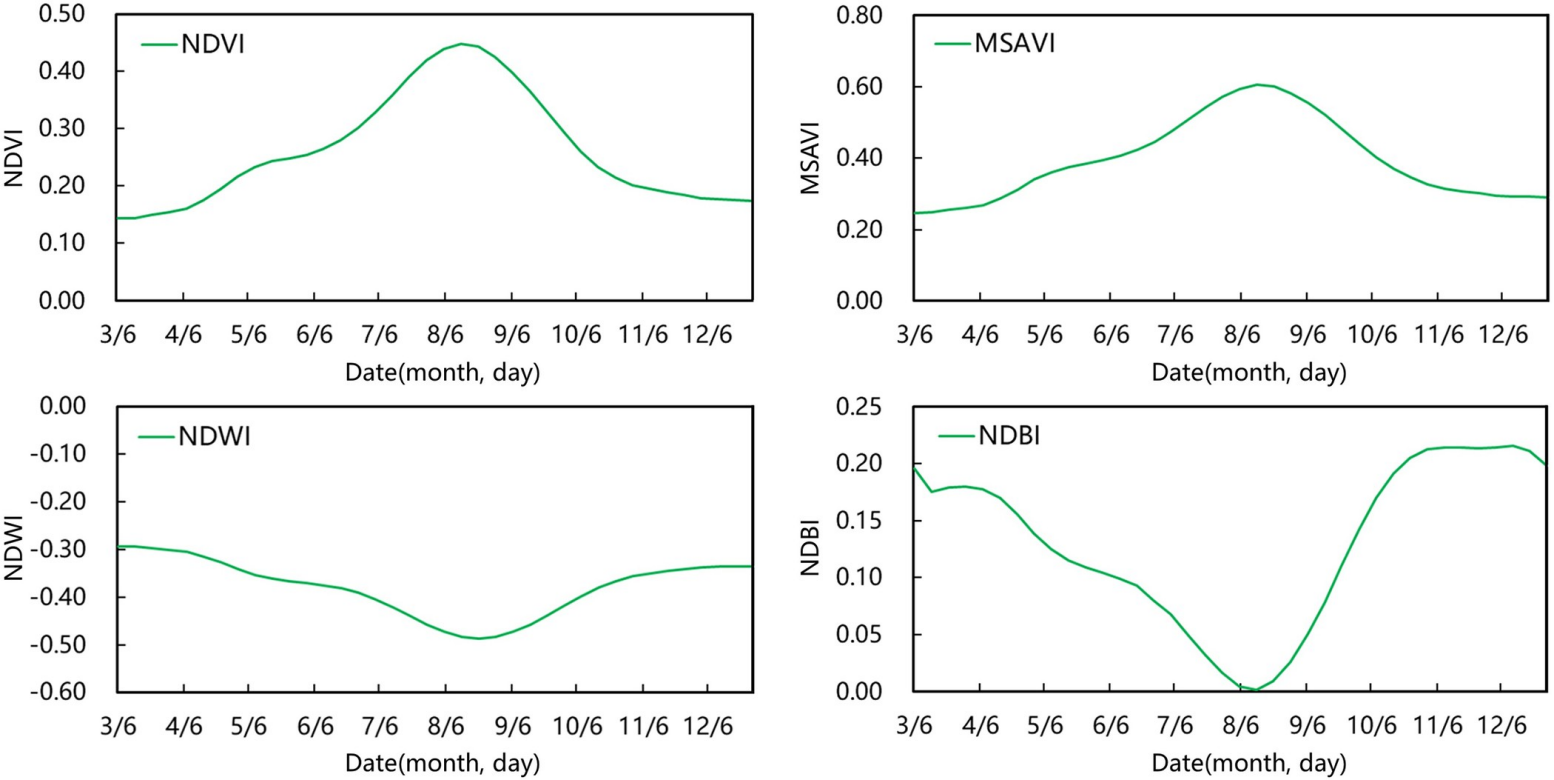
MODIS average NDVI, MSAVI, NDWI, NDBI time series curves for grassland. Note: Abbreviation: NDVI, Normalized Difference Vegetation Index; MSAVI, Modified Soil Adjusted Vegetation Index; NDWI, Normalized Difference Water Index; NDBI, Normalized Difference Built-up Index.

Ensuring the acquisition of valid, high-quality Sentinel-2 images from May to October 2018 was difficult due to the influence of clouds, rain and sunlight. MODIS images were used to calculate four vegetation indices, NDVI, NDWI, NDBI and MSAVI, for determining the optimal time window for synthetic images. This process was performed to reduce data redundancy and enhance remote sensing information whilst ensuring the authenticity and reliability of the data. Four vegetation index mean value curves of NDVI, NDWI, NDBI and MSAVI were plotted to describe the characteristics of grassland changes over time (Fig 4).

The maximum value images with a time window from June 10 to October 24 were synthesised by NDVI and MSAVI. The minimum value image with a time window from June 18 to November 2 was synthesised by NDWI. The minimum value image with a time window from June 10 to October 24 was synthesised by NDBI. If the Sentinel-2 images were synthesised with the time windows of the vegetation indices, then data redundancy would occur, and the data processing speed would be reduced. Therefore, two separate phases of optical median value images were synthesised based on the growth and senescence stages of the grass. The time windows were the growth period from May 9 to August 12 and the senescence period from August 13 to November 2.

### Selection of feature variables

Five features, including the original spectral bands of optical images, vegetation index features and texture features, and the polarisation bands and texture features of SAR images, were selected in this paper (Table 3). The visible band, NIR band and short-wave infrared band of Sentinel-2 images are widely used in vegetation remote sensing, which are of great value for vegetation identification. NDVI plays a crucial role in the classification of vegetation classes [46] and can be used to categorise bare sand and open waterbody very well [47]. MSAVI is very important to increasing the vegetation signal and reduce soil-induced changes simultaneously, thus minimising the effect of soil on vegetation spectra [48]. NDWI provides valuable information on classification characteristics by considering the variability of vegetation water content [49]. NDBI is effective in distinguishing information about vegetation, water bodies and buildings [50].

**Table 3:** Feature variables of the satellite data.

Sensors	Feature types	Feature variable	Abbreviation
Sentinel-1A	Polarization feature	Vertical polarization	VV
Sentinel-2	Spectral feature	Blue	B
		Green	G
		Red	R
		Near Infrared	NIR
		Short-wave InfraredⅠ	SWIRⅠ
		Short-wave InfraredⅡ	SWIRⅡ
	Vegetation Index	Normalized Difference Vegetation Index	NDVI
		Normalized Difference Water Index	NDWI
		Normalized Difference Building Index	NDBI
		Modified Soil Adjusted Vegetation Index	MSAVI
Sentinel-1A Sentinel-2	Texture feature	Angle Secondary Matrix	ASM
		Contrast	CON
		Correlation	COR
		Entropy	ENT

Texture can present the surface or structural properties of an image and is an important feature variable to improve the accuracy of LC classification. However, many texture features cause data redundancy. This paper filtered the texture features whilst retaining the maximum amount of information. Angular second moment (ASM), contrast (CON), correlation (COR) and entropy (ENT) [22] were selected as characteristic variables. ASM represents the coarseness of the texture and the uniformity of grey distribution, CON represents the depth of the texture grooves and the clarity of the image, COR represents the consistency of the texture in the local area and ENT represents the complexity of the texture.

### Experimental scheme

Seven experimental groups were designed, including 14 experimental schemes (Table 4). Single optical imagery and single SAR imagery were designed to complete the grassland distribution information extraction (EXP1-EXP5). This process was performed to investigate the feasibility and the effectiveness of various types of sensor images for grassland mapping. The overall accuracy of the mapping of Sentinel-integrated images (EXP6-EXP7) was compared with the experimental results of the classified mapping of Sentinel-1A SAR images and Sentinel-2 optical images to explore the potential of the integrated images of SAR and optical images in improving the accuracy of grassland mapping.

**Table 4:** Experimental design.

Experimental group	Model	Combination of feature variables	Number of feature variables
EXP1-1	Traditional global classification model	S1 Polarization feature	6
EXP1-2	Rectangular tile classification model		
EXP2-1	Traditional global classification model	S1 Polarization feature + S1 Texture feature	30
EXP2-2	Rectangular tile classification model		
EXP3-1	Traditional global classification model	S2 Spectral feature	12
EXP3-2	Rectangular tile classification model		
EXP4-1	Traditional global classification model	S2 Spectral feature + S2 Vegetation index	16
EXP4-2	Rectangular tile classification model		
EXP5-1	Traditional classification global model	S2 Spectral feature + S2 Vegetation index + S2 Texture feature	32
EXP5-2	Rectangular tile classification model		
EXP6-1	Traditional global classification model	S1 Polarization feature + S1 Texture feature + S2 Spectral feature + S2 Vegetation index	46
EXP6-2	Rectangular tile classification model		
EXP7-1	Traditional global classification model	S1 Polarization feature + S1 Texture feature + S2 Spectral feature + S2 Vegetation index + S2 Texture feature	62
EXP7-2	Rectangular tile classification model		

Note: Abbreviation: S1: Sentinel-1A; S2: Sentinel-2.

**Fig 5:**
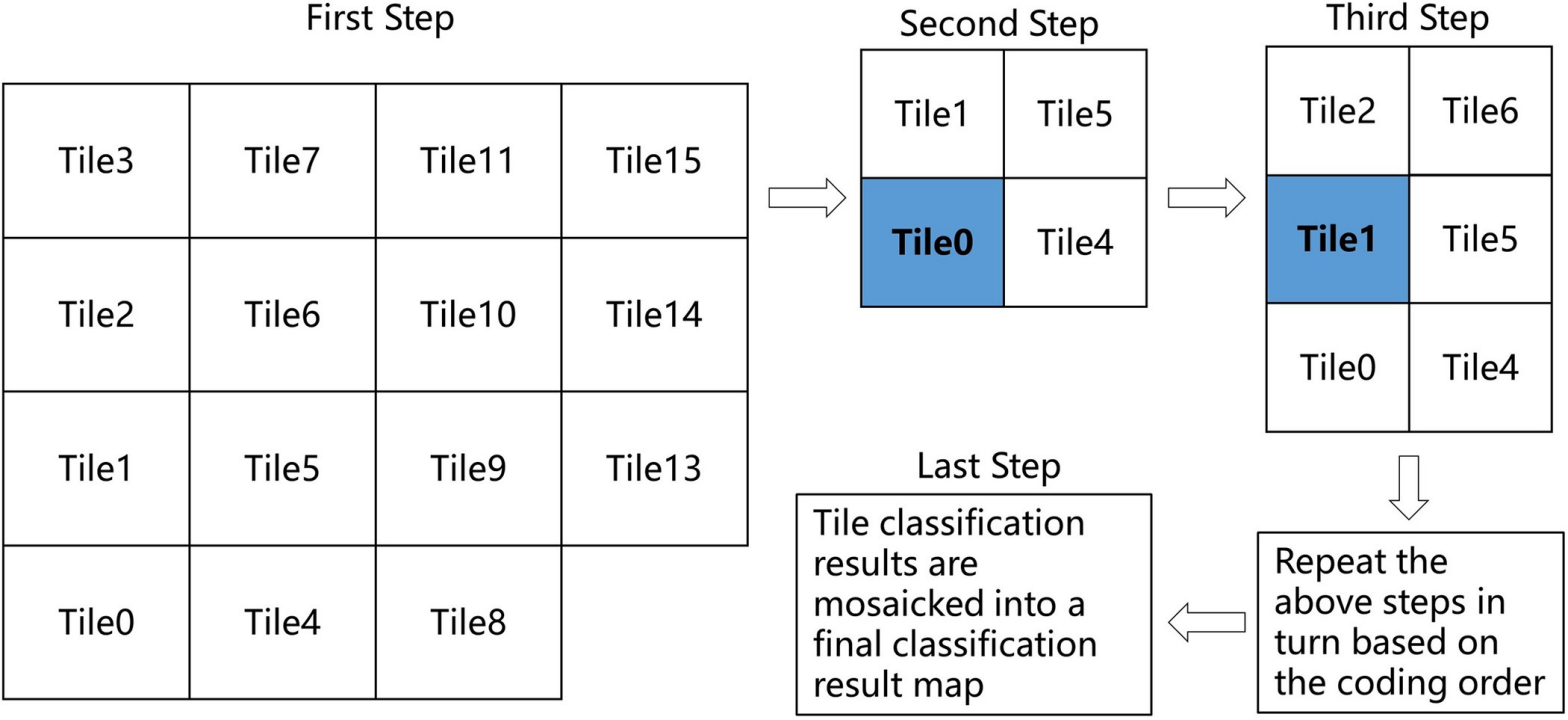
Schematic of the random forest classifier operation based on the rectangular tile classification model. Note: First Step: 0–15 represents the coding order of the tiles. Second Step: The blue tile represents the central tile and the white tiles represent the adjacent tiles.

LC in the highly heterogeneous areas of the landscape exhibited complex, fragmented characteristics with changes in geospatial location, especially in the study area where geographic longitudes and latitudes span large distances. The study area was divided into 15 tiles (Fig 5), and LCs in each tile possessed similar spectral characteristics to address the effects of geospatial location differences on spectral features. This method obeys the first law of geography and can effectively reduce the identification error caused by different spectral characteristics and phenological characteristics of the same LC type in the study area.

### Rectangular tile classification model

**Fig 6:**
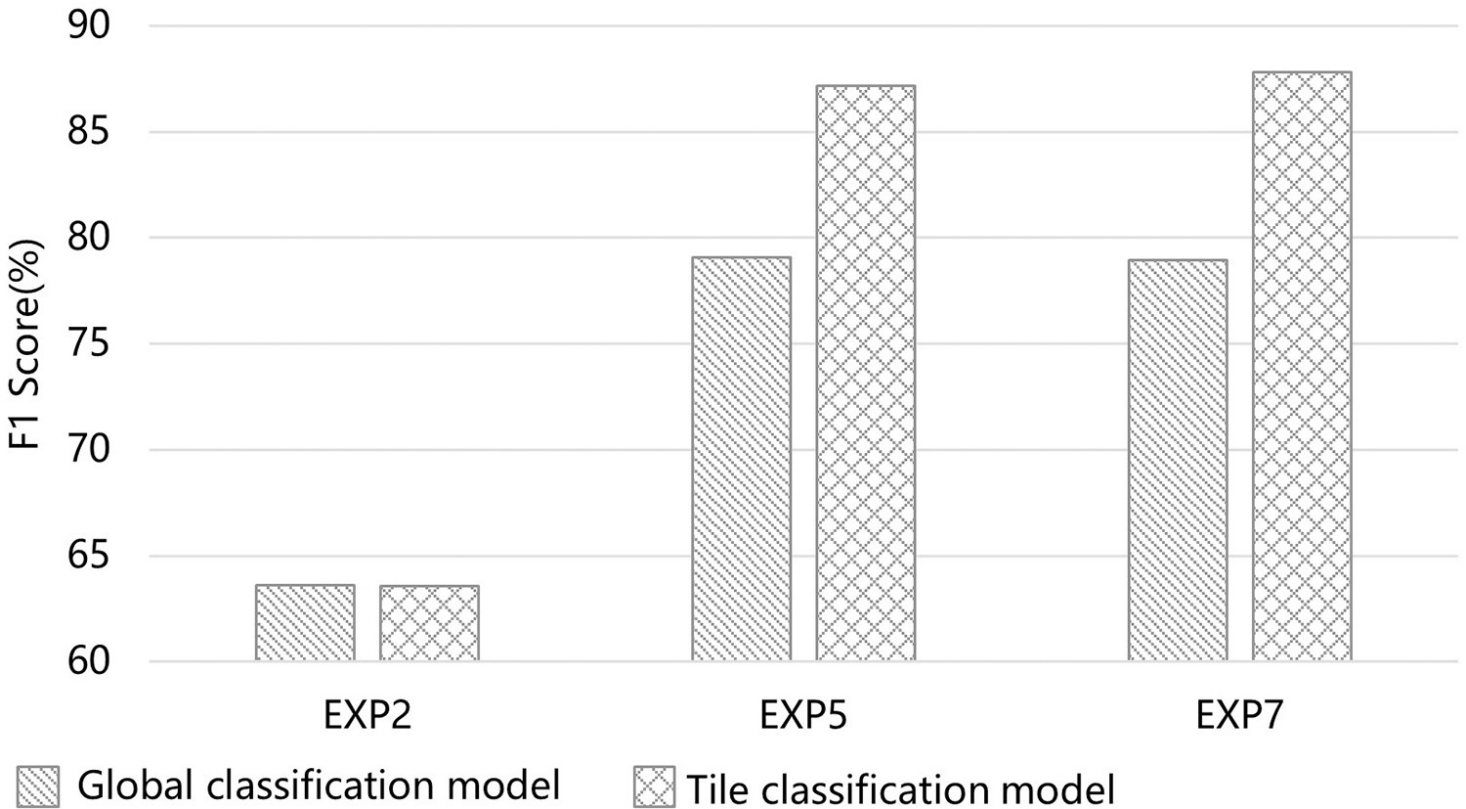
Comparisons among EXP2, EXP5, and EXP7 by F1 score. Note: EX2: S1 Polarization feature + S1 Texture feature; EXP5: S2 Spectral feature + S2 Vegetation index + S2 Texture feature; EXP7: S1 Polarization feature + S1 Texture feature + S2 Spectral feature + S2 Vegetation index + S2 Texture feature. The F1 score is a weighted average of the mapping accuracy and user accuracy.

In this paper, the study area was divided into 15 equal-area rectangular tiles, the images within each tile were independently classified based on the RF algorithm, and all tile classification results were integrated into a final classification result map (Fig 6). During the model training, the tile order was cycled sequentially. The rectangular tile classification model utilised the training samples of the central tile and the surrounding adjacent tiles to participate in the RF model training jointly. Compared with the rectangular tile classification model, the traditional global classification model utilised all the training samples in the study area as the construction of the RF algorithm model at the same time. The tile classification model reduced the error of the variation of spectral features amongst different regions [37] and ensured the random uniform distribution of different LCs in Ordos. Sample points of approximately six major LC types within each rectangular tile were randomly selected as training samples. However, each tile had a different number of samples within it to balance the proportion of small area LC types, such as cultivated land, forest land, waterbody and building. All samples were selected uniformly across the study area to avoid spatial correlation.

### RF algorithm

The RF algorithm, which consists of multiple decision trees and is a supervised classification ensemble learning algorithm [51], was proposed by Breiman [52]. The number of feature variables and decision trees needs to be set when using this algorithm in the GEE environment. With the same number of feature variables, the number of decision trees is increased sequentially, and the overall accuracy is improved with the increase in the number of decision trees. The accuracy reaches saturation without varying with the number of decision trees. Referring to previous studies and combining multiple experiments, the number of decision trees in this paper was set to 50 [25].

### Accuracy evaluation

The other approximately 30% of the sample data were used as validation set for classification accuracy verification. The error matrix and its accuracy indices are used to evaluate the extraction results quantitatively, including overall accuracy (OA), kappa coefficient, producer accuracy (PA), user accuracy (UA) and F1 score [53, 54]. OA and kappa coefficient are the overall metrics used to evaluate the classification results. PA and UA are indicators to measure the accuracy of feature classification, and can reflect the quality of feature classification from different aspects. The F1 score is a measure of the accuracy of a binary classification model and is a weighted average of the PA and UA of the model. The F1 scores were used to compare and analyse the mapping results of this study with 30 m Landsat-derived land use/ land cover (LULC) maps (CNLUCC-2018 and WHCLCD-2018).

## Results

### Accuracy of experimental schemes

The grassland distribution information was extracted by using different experimental schemes, and the OA, kappa coefficient, PA, UA and F1 score of different schemes were calculated. The results in Table 5 show OA improved with the increase in the number of feature variables when using Sentinel-1A and Sentinel-2 images alone for grassland mapping. Based on the rectangular tile classification model, the F1 Scores showed a slight increase as the dimensionality of the classification features increased, which can be observed when comparing EXP3-2, EXP4-2, EXP5-2, EXP6-2, and EXP7-2. In particular, the F1 score in EXP7-2 on the rectangular tile classification model reached the maximum of 89.24%. When using Sentinel-integrated images to extract grassland, the OA was improved in varying degrees compared with that of a single sensor image. For each experimental group, all five grassland mapping accuracy metrics of the rectangular tile classification model were improved to a greater extent compared with the traditional global classification model. Comparisons of the mapping results of EXP2, EXP5 and EXP7 based on the traditional global classification model and the rectangular tile classification model were evaluated by F1 scores (Fig 6).

**Table 5:** Accuracy of experimental schemes.

Experimental group	Overall accuracy/%	Kappa coefficient	Producer accuracy /%	User Accuracy/%	F1 Score/%
EXP1-1	51.45	0.3381	72.32	48.61	58.14
EXP1-2	53.52	0.3687	74.36	48.68	58.84
EXP2-1	58.34	0.4302	80.73	52.41	63.56
EXP2-2	60.70	0.4531	80.66	56.50	66.45
EXP3-1	72.85	0.6384	87.32	67.80	76.33
EXP3-2	85.08	0.8045	91.75	81.64	86.40
EXP4-1	75.62	0.6752	88.78	68.61	77.40
EXP4-2	86.38	0.8211	92.18	82.45	87.05
EXP5-1	76.14	0.6827	89.51	70.85	79.09
EXP5-2	86.53	0.8264	91.31	84.51	87.78
EXP6-1	76.18	0.6826	89.02	68.87	77.66
EXP6-2	87.01	0.8291	92.62	84.79	88.53
EXP7-1	77.00	0.6942	89.27	70.79	78.96
EXP7-2	88.23	0.8461	93.11	85.68	89.24

**Fig 7:**
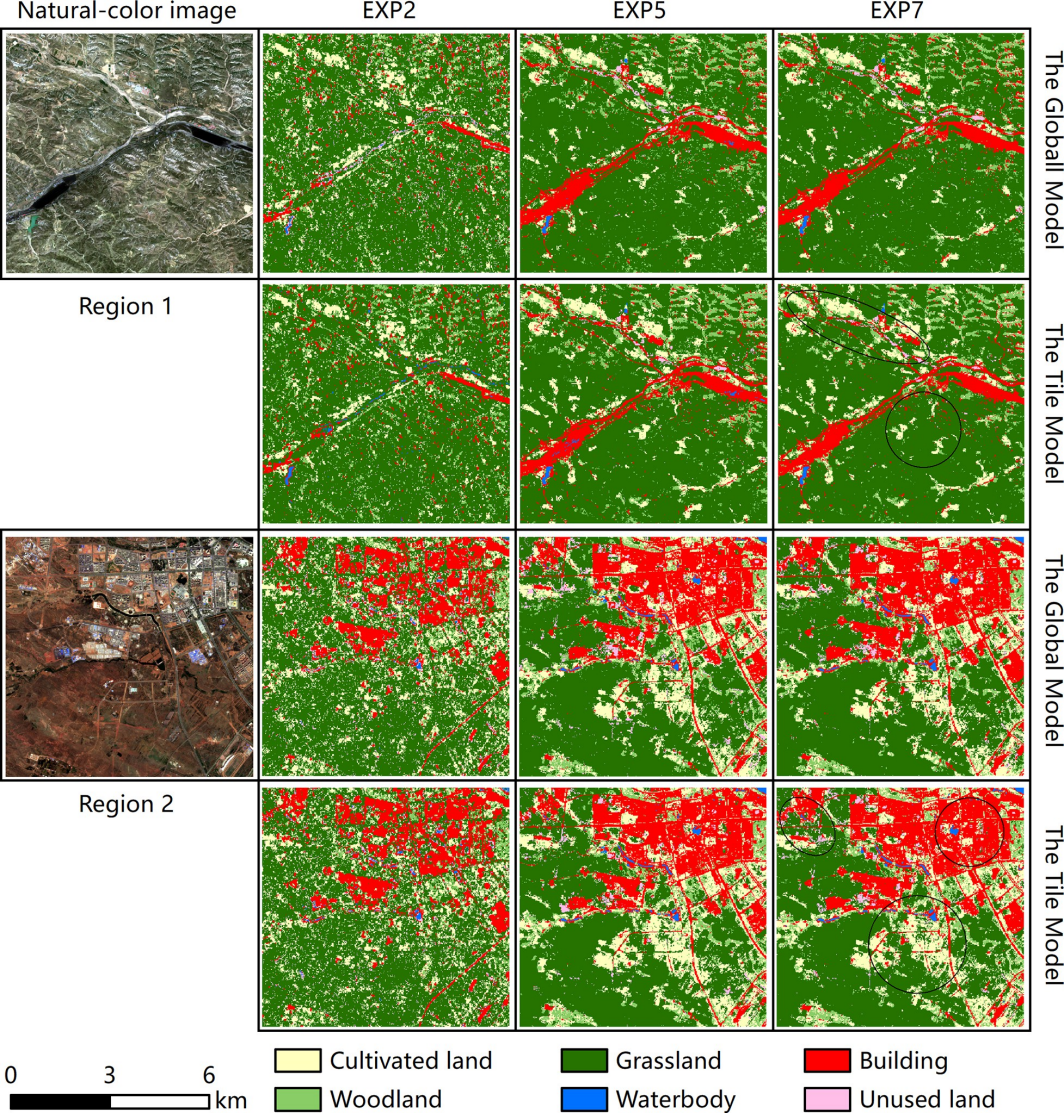
Detailed comparisons among classification result from EXP2, EXP5, and EXP7 by spatial patterns in two regions.

The detailed comparisons of three LULC maps between EXP2, EXP5 and EXP7 are shown in Fig 7. Two complex typical small regions were segmented for comparative analysis based on the traditional global classification model and the rectangular tile classification model. Region 1 (110°42′33″ E, 39°36′35″ N) is in the eastern gully area of the study area and is a typical grassland zone. Region 2 (109°43′08″ E, 39°33′05″ N) is in the urban area of Ejin Horo Banner. In region 1, the classification results of EXP2 and EXP5 had more building speckle noise based on the rectangular tile and traditional global classification models, but the classification results of EXP7 had considerably less building speckle noise. In EXP7, the classification results based on the rectangular tile classification model were clearer and neater, and the whole area was more consistent with the actual LC. In Region 2, the classification results of EXP2 clearly showed the misclassification of buildings, cultivated lands and grasslands. EXP5 had a part of cultivated land and grassland patches misclassified as unused land based on the rectangular tile and traditional global classification models, and the classification results of EXP7 were improved to some extent. The classification results of EXP7 were based on the rectangular tile classification model, and the classification results within the whole area were more consistent with the actual LC.

### Grassland spatial distribution

From EXP7-2 based on Sentinel-integrated images, the spatial distribution map of Ordos grassland with a spatial resolution of 20 m was drawn by using the rectangular tile classification model to extract the grassland distribution information of the study area. Comparisons between the classification result from EXP7-2, CNLUCC-2018 (Kuang et al., 2022) and WHCLCD-2018 (Yang and Huang, 2021) by spatial patterns indicate most of the grasslands were correctly classified, grasslands in the region were mainly distributed in the central and south-western parts of the study area, and the vegetation was relatively sparse in the Kubuqi Sand in the north and the Maowusu Sand in the southeast.

The error matrix from EXP7-2 was constructed by using the classification results of grassland, woodland, cultivated land, waterbody, building and unused land in the study area. The OA, kappa coefficient, PA, UA and F1 score were calculated. The results are shown in Table 6. The PA, UA and F1 score of the grassland were 93.11%, 85.68% and 89.24% respectively.

**Table 6:** Confusion matrix for EXP7-2 of the grassland distribution map in Ordos, China.

Land cover	Classification results								
	Cultivated land	Woodland	Grassland	Waterbody	Building	Unused land	Total	User accuracy	F1 Score
Cultivated land	523	14	22	1	3	14	577	90.64%	92.00%
Woodland	12	191	17	0	2	4	226	84.51%	78.12%
Grassland	13	35	784	0	4	79	915	85.68%	89.24%
Waterbody	1	2	0	159	0	1	283	97.55%	98.15%
Building	6	2	0	0	158	3	279	93.49%	93.21%
Unused land	5	19	19	1	3	298	345	86.38%	80.11%
Total	560	263	842	161	170	399	2395		
Producer accuracy	93.39%	77.62%	93.11%	98.76%	92.94%	74.69%			

Note: The value in the middle of the confusion matrix represents the number of pixels.

### Comparisons between the Derived LULC map, CNLUCC-2018 and WHCLCD-2018 of Ordos

**Fig 8:**
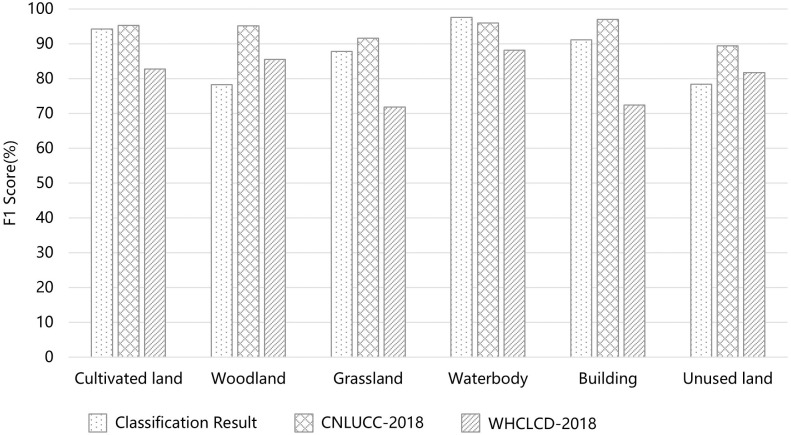
Comparisons among classification result from EXP7-2, CNLUCC-2018, and WHCLCD-2018 by F1 score.

The obtained 20 m Sentinel-2 derived LULC map from EXP7-2 in this paper and the 30 m Landsat-derived LULC maps (CNLUCC-2018 and WHCLCD-2018) were compared in terms of F1 score (Fig 8). Prior to the comparative analysis, CNLUCC-2018 and WHCLCD-2018 were reclassified into a unified LC criteria system. The F1 score for grassland was lower than CNLUCC-2018 and higher than WHCLCD-2018.

**Fig 9:**
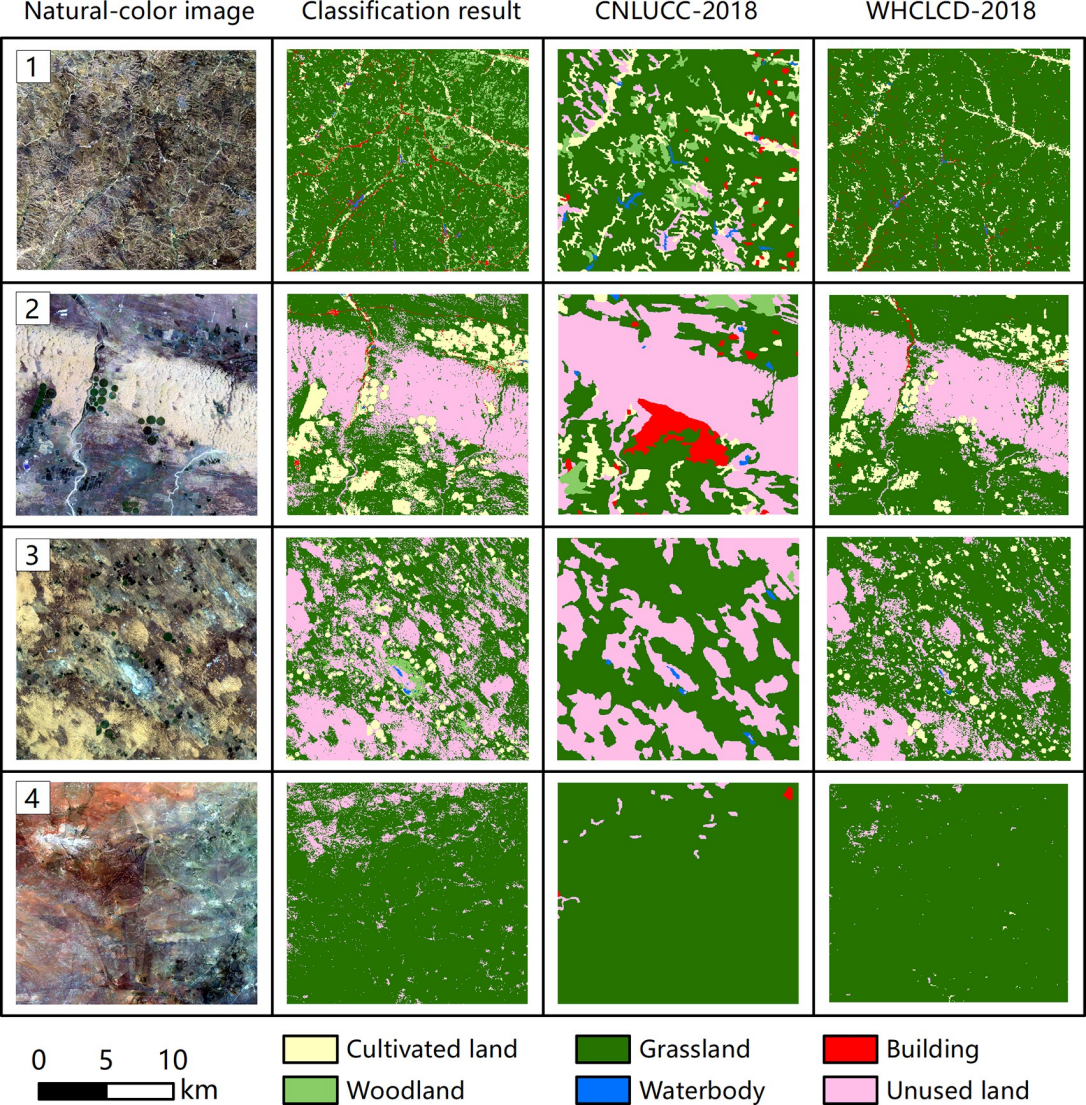
Detailed comparisons among classification result from EXP7-2, CNLUCC-2018, and WHCLCD-2018 by spatial patterns in four regions.

The detailed comparisons of three LULC maps are shown in Fig 9. Four typical small regions were segmented from the study area for detailed comparative analysis. Region 3 (110°56′11″ E, 39°48′53″ N) is in the eastern gully area of the study area and is a typical grassland zone. Region 4 (109°18′51″ E, 40°21′22″ N) is in the northern part of the study area in the Kubuqi Sand, which is a desert grassland zone. Region 5 (107°58′10″ E, 38°08′04″ N) is in the south-eastern part of the study area in the Maowusu Sand, which is the desert grassland zone. Region 6 (107°24′10″ E, 39°39′13″ N) is in the undulating plateau zone in the western part of the study area, which is the grassland desertification zone. In region 3, our results were more consistent with the actual LC, and the red line feature roads were correctly extracted. The road information in CNLUCC-2018 and WHCLCD-2018 was not shown. The area of unused land and building in CNLUCC-2018 was large. The woodland area in WHCLCD-2018 was small. In region 4, our results were more consistent with WHCLCD-2018, where fine-grazed grassland patches and cultivated land patches in the sand were correctly extracted. A larger red building patch was clearly misclassified in CNLUCC-2018, and a larger number of cultivated land patches were omitted. In region 5, our results and WHCLCD-2018 were more consistent with the actual LC. The area of unused land in CNLUCC-2018 was remarkably larger, and all cultivated patches were omitted. In region 6, our results were more consistent with the actual LC. However, one red building patch was missed. The red building patch in CNLUCC-2018 was extracted correctly, but many fine unused land patches were missed. The red building patch in WHCLCD-2018 was also missed.

## Discussion

Comparing the grassland mapping accuracy of EXP3-2, EXP4-2, and EXP5-2 by F1 score, our results showed that the integration of vegetation index features and texture features improved the accuracy of grassland mapping. In EXP4-2, the inclusion of vegetation indices bands resulted in a 0.75% improvement in grassland mapping accuracy compared to EXP3-2. Similarly, in EXP5-2, the addition of texture features led to a further improvement of 0.84% in grassland mapping accuracy compared to EXP4-2. Two main reasons explain the difficulty of extracting information from grasslands in Ordos arid zone: Firstly, shrubs and herbs are the main vegetation types, vegetation cover is low, and vegetation is scattered in the arid desert grassland subzone and grassland sandy subzone [55]. Secondly, bare sand within unused land has a substantial effect on the spectral behaviour of the canopy of shrub and herbaceous vegetation with sparse vegetation cover [56]. To reduce the adverse effects of low vegetation cover and bare sand, Ge et al. [47] introduced NDVI, MSAVI, NDWI and NDBI indices to improve the accuracy of LC mapping in arid desert oases effectively. Shafizadeh-Moghadam et al. [24] found that incorporating texture features could be beneficial in enhancing overall accuracy in study of LC mapping of six Middle Eastern countries in the Tigris-Euphrates River Basin.

Integration of Sentinel-1A SAR imagery to Sentinel-2 images can improve the accuracy of grassland mapping. Comparing the grassland mapping accuracy of EXP5-2 and EXP7-2, the mapping accuracy of Sentinel-integrated images was improved by 1.66% compared with Sentinel-2 images in terms of F1 score. Previous studies [27, 57] showed the use of integrated imagery to map the spatial distribution of grasslands is more advantageous. Their research results showed integrated images help improve the accuracy of LC classification in grassland areas. When optical images are affected by clouds and rain resulting in less optical image coverage in the study area, it can greatly limit the remote sensing land classification mapping in the study area. In this case, SAR images can be used as supplementary data for optical images because the microwave of the SAR sensor can penetrate the canopy of vegetation to obtain information on vegetation coverage, vegetation moisture content and soil background. In the Ordos Plateau, cloudy weather is concentrated from February 9 to September 15 every year. Therefore, LC classification using Sentinel-integrated images in this paper is feasible and valuable.

In our paper, the grassland mapping accuracy of all experimental groups based on the rectangular tile classification model was higher than that of the traditional global classification model. For large-scale LC mapping, a certain type of LC can easily be mistaken for other types of LC due to the influence of spectral features, for example, woodland and grassland [37]. Domestic and international scholars have recently conducted some research to improve the accuracy of remote sensing mapping on large scale. Bartalev et al. [58] proposed a new locally adaptive classification method (locally adaptive global mapping algorithm) for large-scale LC mapping using remote sensing data, which was the grid-based supervised image classification using classes’ features estimated locally in classified pixels’ surrounding from spatially distributed reference data. They found the traditional global classification model accuracy was 81.8%, and the local model accuracy was 96.8%. Although the classification algorithm improved the classification accuracy remarkably, the limited data processing capacity of the local platform affected the mapping efficiency.

The emergence of GEE provides a feasible means to overcome this challenge. GEE greatly facilitates researchers worldwide to conduct research on large-scale LC classification and dynamic monitoring of land use change. Zhang et al. [37] used Sentinel-2 time series, tile-based image classification and GEE to generate high-resolution LC in Madagascar automatically. They found the average PA of the traditional global classification method was 84.8%, and the PA of the tile classification method was only 88.2%, which indicated the tile classification method can remarkably improve the accuracy of classification mapping and reduce misclassification amongst LC types. Xuan et al. [59] mapped crop type in Northeast China during 2013–2021 using automatic sampling and tile-based image classification, but they did not conduct a comparative analysis of the classification accuracy of the tile classification model and the traditional global classification model. The tile classification model based on RF classifier considered the similarity and specificity of vegetation structure within climatic subzones. Inspired by the above studies, our remote sensing mapping study of Ordos grassland was based on the rectangular tile classification model and integrated image on the GEE platform. In our paper, the F1 score of grassland in EXP7-2 on the rectangular tile classification model reached the maximum value of 89.24%, which was improved by 13.02% compared with EXP7-1 on the traditional global classification model. The adoption of the rectangular tile classification model in large-scale LC classification mapping was conducive to remarkably improving the mapping accuracy. This conclusion was consistent with the findings of Zhang et al. [37] in LC mapping in Madagascar, which provided a new idea for subsequent LC mapping of highly heterogeneous landscapes on a large scale.

## Limitations and recommendation

Although the accuracy of grassland mapping based on the rectangular tile classification model was remarkably improved than the traditional global classification model, the paper had limitations. Firstly, the optimal scale regarding the rectangular tile classification model was not explored in depth. Only rectangular tiles were utilised, and other shapes of tiles were ignored. Secondly, the spectral differences between images were not explored during image synthesis, which may increase the error of the experimental results because the classifier treats excessive differences as noise. Thirdly, Sentinel-2 images using time window synthesis can reduce data redundancy, but the information of Sentinel-2 time-series images cannot be fully exploited. Sentinel-2 monthly synthetic images can be considered to enhance the utilisation of image information. Finally, only one algorithm of RF was utilised. In future study, we will focus on enhancing the construction of sample datasets and vegetation index feature datasets. Additionally, we will strive to improve the algorithms by incorporating soft classification algorithms and deep learning algorithms into the study of grassland mapping in arid zones.

## Conclusion

In this paper, the rectangular tile classification model based on RF algorithm and Sentinel-integrated imagery on GEE platform was utilised to map the spatial distribution of Ordos grassland in 2018. Comparing the grassland mapping accuracy of EXP3-2, EXP4-2, and EXP5-2 by F1 score, our results showed that the integration of vegetation index features and texture features improved the accuracy of grassland mapping. Integration of Sentinel-1A SAR imagery with Sentinel-2 images can improve the accuracy of grassland mapping. In all seven experimental groups, the rectangular tile classification model was found to improve overall accuracy (OA) by 1.20% to 13.99% compared to the traditional global classification model. The adoption of the rectangular tile classification model in large-scale grassland mapping was conducive to remarkably improving the mapping accuracy in arid zone. This paper and its results provide some new viewpoints and guidance for remote sensing mapping in highly heterogeneous landscapes areas on a large scale.

## Supporting information

S1 File The vector boundaries of China’s provinces, the nine-dash line and Ordos. (RAR)

S2 File Remote sensing data of Region 1, Region 2, Region 3, Region 4, Region 5 and Region 6. (RAR)

S3 File EXP7-2, CNLUCC-2018 and WHCLCD-2018. (RAR)

## References

[1] Wei P, Xu L, Pan X, Hu Q, Li Q, Zhang X, et al. Spatio-temporal variations in vegetation types based on a climatic grassland classification system during the past 30 years in Inner Mongolia, China. Catena. 2020;185:104298. doi: 10.1016/j.catena.2019.104298

[2] Xu Y, Dong K, Jiang M, Liu Y, He L, Wang J, et al. Soil moisture and species richness interactively affect multiple ecosystem functions in a microcosm experiment of simulated shrub encroached grasslands. Science of The Total Environment. 2022;803:149950. Epub 2021/09/07. doi: 10.1016/j.scitotenv.2021.149950 . 34487904

[3] Han P, Zhao X, Dong Z, Yan Y, Niu J, Zhang Q. A new approach for the classification of grassland utilization in Inner Mongolia—based on ecological sites and state-and-transition models. Ecological Indicators. 2022;137:108733. doi: 10.1016/j.ecolind.2022.108733

[4] Ali I, Cawkwell F, Dwyer E, Barrett B, Green S. Satellite remote sensing of grasslands: from observation to management. Journal of Plant Ecology. 2016;9(6):649–71. doi: 10.1093/jpe/rtw005

[5] Phiri D, Simwanda M, Salekin S, Nyirenda V, Murayama Y, Ranagalage M. Sentinel-2 Data for Land Cover/Use Mapping: A Review. Remote Sensing. 2020;12(14):2291. doi: 10.3390/rs12142291

[6] Zhang Z, Wei M, Pu D, He G, Wang G, Long T. Assessment of Annual Composite Images Obtained by Google Earth Engine for Urban Areas Mapping Using Random Forest. Remote Sensing. 2021;13(4):748. doi: 10.3390/rs13040748

[7] Phan TN, Kuch V, Lehnert LW. Land Cover Classification using Google Earth Engine and Random Forest Classifier—The Role of Image Composition. Remote Sensing. 2020;12(15):2411. doi: 10.3390/rs12152411

[8] Mellor A, Boukir S, Haywood A, Jones S. Exploring issues of training data imbalance and mislabelling on random forest performance for large area land cover classification using the ensemble margin. ISPRS Journal of Photogrammetry and Remote Sensing. 2015;105:155–68. doi: 10.1016/j.isprsjprs.2015.03.014

[9] Wessels K, van den Bergh F, Roy D, Salmon B, Steenkamp K, MacAlister B, et al. Rapid Land Cover Map Updates Using Change Detection and Robust Random Forest Classifiers. Remote Sensing. 2016;8(11):888. doi: 10.3390/rs8110888

[10] Reinke K, Jones S. Integrating vegetation field surveys with remotely sensed data. Ecological Management & Restoration. 2006;7(S1):18–23. doi: 10.1111/j1442-8903.2006.00287.x

[11] Badreldin N, Prieto B, Fisher R. Mapping Grasslands in Mixed Grassland Ecoregion of Saskatchewan Using Big Remote Sensing Data and Machine Learning. Remote Sensing. 2021;13(24):4972. doi: 10.3390/rs13244972

[12] Gong P, Wang J, Yu L, Zhao Y, Zhao Y, Liang L, et al. Finer resolution observation and monitoring of global land cover: first mapping results with Landsat TM and ETM+ data. International Journal of Remote Sensing. 2012;34(7):2607–54. doi: 10.1080/01431161.2012.748992

[13] Chen J, Chen J, Liao A, Cao X, Chen L, Chen X, et al. Global land cover mapping at 30m resolution: A POK-based operational approach. ISPRS Journal of Photogrammetry and Remote Sensing. 2015;103:7–27. doi: 10.1016/j.isprsjprs.2014.09.002

[14] Yang J, Huang X. The 30 m annual land cover dataset and its dynamics in China from 1990 to 2019. Earth System Science Data. 2021;8(13):3907–25. doi: 10.5194/essd-2021-7

[15] Kuang W, Zhang S, Du G, Yan C, Wu S, Li R. Remotely sensed mapping and analysis of spatio-temporal patterns of land use change across China in 2015–2020. Acta Geographica Sinica (Chinese). 2022;77(05):1056–71. doi: 10.11821/dlxb202205002

[16] Campos JC, Brito JC. Mapping underrepresented land cover heterogeneity in arid regions: The Sahara-Sahel example. ISPRS Journal of Photogrammetry and Remote Sensing. 2018;146:211–20. doi: 10.1016/j.isprsjprs.2018.09.012

[17] Zeng H, Wu B, Wang S, Musakwa W, Tian F, Mashimbye ZE, et al. A Synthesizing Land-cover Classification Method Based on Google Earth Engine: A Case Study in Nzhelele and Levhuvu Catchments, South Africa. Chinese Geographical Science. 2020;30(3):397–409. doi: 10.1007/s11769-020-1119-y

[18] Hou M, Ge J, Xiu Y, Meng B, Liu J, Feng Q, et al. The urgent need to develop a new grassland map in China: based on the consistency and accuracy of ten land cover products. Sci China Life Sci. 2023;66(2):385–405. Epub 2022/08/31. doi: 10.1007/s11427-021-2143-3 . 36040706

[19] Liu T, Han P, Guo ML, Dong JJ, Ren JB, Tian YW, et al. Extracting Spatial Distribution of Rainfed Artificial Alfalfa Grassland Based on Multi-Temporal Remote Sensing Data. Chinese Journal of Grassland (Chinese). 2018;40(06):56–63. doi: 10.16742/j.zgcdxb.2018-06-08

[20] Herrero HV, Southworth J, Bunting E, Kohlhaas RR, Child B. Integrating Surface-Based Temperature and Vegetation Abundance Estimates into Land Cover Classifications for Conservation Efforts in Savanna Landscapes. Sensors (Basel). 2019;19(16):3456. Epub 2019/08/10. doi: 10.3390/s19163456 ; PubMed Central PMCID: PMC6720620. 31394848 PMC6720620

[21] Sun Z, Xu R, Du W, Wang L, Lu D. High-Resolution Urban Land Mapping in China from Sentinel 1A/2 Imagery Based on Google Earth Engine. Remote Sensing. 2019;11(7):752. doi: 10.3390/rs11070752

[22] Ma Z, Liu C, Xue H, Li J, Fang X, Zhou J. Identification of Winter Wheat by Integrating Active and Passive Remote Sensing Data Based on Google Earth Engine Platform. Transactions of the Chinese Society for Agricultural Machinery (Chinese). 2021;52(09):195–205. doi: 10.6041/j.issn.1000-1298 2021.09.023.

[23] Taravat A, Wagner M, Oppelt N. Automatic Grassland Cutting Status Detection in the Context of Spatiotemporal Sentinel-1 Imagery Analysis and Artificial Neural Networks. Remote Sensing. 2019;11(6):711. doi: 10.3390/rs11060711

[24] Shafizadeh-Moghadam H, Khazaei M, Alavipanah SK, Weng Q. Google Earth Engine for large-scale land use and land cover mapping: an object-based classification approach using spectral, textural and topographical factors. GIScience & Remote Sensing. 2021;58(6):914–28. doi: 10.1080/15481603.2021.1947623

[25] Li C, Chen W, Wang Y, Wang Y, Ma C, Li Y, et al. Mapping Winter Wheat with Optical and SAR Images Based on Google Earth Engine in Henan Province, China. Remote Sensing. 2022;14(2):284. doi: 10.3390/rs14020284

[26] Hong G, Zhang A, Zhou F, Brisco B. Integration of optical and synthetic aperture radar (SAR) images to differentiate grassland and alfalfa in Prairie area. International Journal of Applied Earth Observation and Geoinformation. 2014;28:12–9. doi: 10.1016/j.jag.2013.10.003

[27] Samrat A, Devy MS, Ganesh T. Delineating fragmented grassland patches in the tropical region using multi-seasonal synthetic aperture radar (SAR) and optical satellite images. International Journal of Remote Sensing. 2021;42(10):3938–54. doi: 10.1080/01431161.2021.1881181

[28] de Oliveira Santos CLM, Lamparelli RAC, Dantas Araújo Figueiredo GK, Dupuy S, Boury J, Luciano ACdS, et al. Classification of Crops, Pastures, and Tree Plantations along the Season with Multi-Sensor Image Time Series in a Subtropical Agricultural Region. Remote Sensing. 2019;11(3):334. doi: 10.3390/rs11030334

[29] Shih H-c Stow DA, Tsai YH. Guidance on and comparison of machine learning classifiers for Landsat-based land cover and land use mapping. International Journal of Remote Sensing. 2018;40(4):1248–74. doi: 10.1080/01431161.2018.1524179

[30] Heydari SS. Large Area Land Cover Mapping Using Deep Neural Networks and Landsat Time-Series Observations: State University of New York College of Environmental Science and Forestry; 2021.

[31] Zhang C, Zhang H, Tian S. Phenology-assisted supervised paddy rice mapping with the Landsat imagery on Google Earth Engine: Experiments in Heilongjiang Province of China from 1990 to 2020. Computers and Electronics in Agriculture. 2023;212. doi: 10.1016/j.compag.2023.108105

[32] Huang H, Wang J, Liu C, Liang L, Li C, Gong P. The migration of training samples towards dynamic global land cover mapping. ISPRS Journal of Photogrammetry and Remote Sensing. 2020;161:27–36. doi: 10.1016/j.isprsjprs.2020.01.010

[33] Freeman EA, Moisen GG, Frescino TS. Evaluating effectiveness of down-sampling for stratified designs and unbalanced prevalence in Random Forest models of tree species distributions in Nevada. Ecological Modelling. 2012;233:1–10. doi: 10.1016/j.ecolmodel.2012.03.007

[34] Rayhan F, Ahmed S, Mahbub A, Jani R, Shatabda S, Farid DM, editors. CUSBoost: Cluster-Based Under-Sampling with Boosting for Imbalanced Classification. IEEE International Conference on Computational Systems and Information Technology for Sustainable Solutions; 2017 2017/1/1; Ithaca: IEEE.

[35] Naboureh A, Li A, Bian J, Lei G, Amani M. A Hybrid Data Balancing Method for Classification of Imbalanced Training Data within Google Earth Engine: Case Studies from Mountainous Regions. Remote Sensing. 2020;12(20):3301. doi: 10.3390/rs12203301

[36] Gu Q, Tian J, Li X, Jiang S. A novel Random Forest integrated model for imbalanced data classification problem. Knowledge-Based Systems. 2022;250:109050. doi: 10.1016/j.knosys.2022.109050

[37] Zhang M, Huang H, Li Z, Hackman KO, Liu C, Andriamiarisoa RL, et al. Automatic High-Resolution Land Cover Production in Madagascar Using Sentinel-2 Time Series, Tile-Based Image Classification and Google Earth Engine. Remote Sensing. 2020;12(21):3663. doi: 10.3390/rs12213663

[38] Huang L, Zhou W, Li H, Zhou F, Yang H. Effect of Land Use/Cover Change on Grassland NPP in Grassland Ecosystem of Ordos City. Bulletin of Soil and Water Conservation (Chinese). 2018;38(04):46–52+9+2. doi: 10.13961/j.cnki.stbctb.2018.04.008

[39] Zhang Y, Meng J, Zhou T. Dynamic analysis of landscape structure and function in Erdos during 1988–2000. Journal of Arid Land Resources and Environment (Chinese). 2009;23(05):49–55. doi: 10.13448/j.cnki.jalre.2009.05.001

[40] Statistics OBO. Ordos City National Economy in 2021 and Social Development Statistical Bulletin Ordos, China 2022 [updated 2022/3/21; cited 2022 0012/5/1]. Available from: http://tjj.ordos.gov.cn/dhtjsj/tjgb_78354/202203/t20220321_3165031.html.

[41] Guo Q, Fu B, Shi P, Cudahy T, Zhang J, Xu H. Satellite Monitoring the Spatial-Temporal Dynamics of Desertification in Response to Climate Change and Human Activities across the Ordos Plateau, China. Remote Sensing. 2017;9(6). doi: 10.3390/rs9060525

[42] Balzter H, Cole B, Thiel C, Schmullius C. Mapping CORINE Land Cover from Sentinel-1A SAR and SRTM Digital Elevation Model Data using Random Forests. Remote Sensing. 2015;7(11):14876–98. doi: 10.3390/rs71114876

[43] Drusch M, Del Bello U, Carlier S, Colin O, Fernandez V, Gascon F, et al. Sentinel-2: ESA’s Optical High-Resolution Mission for GMES Operational Services. Remote Sensing of Environment. 2012;120:25–36. doi: 10.1016/j.rse.2011.11.026

[44] Barnes WL, Xiong X, Salomonson VV. Status of terra MODIS and aqua modis. Advances in Space Research. 2003;32(11):2099–106. doi: 10.1016/s0273-1177(03)90529-1

[45] Chen Y, Cao R, Chen J, Liu L, Matsushita B. A practical approach to reconstruct high-quality Landsat NDVI time-series data by gap filling and the Savitzky–Golay filter. ISPRS Journal of Photogrammetry and Remote Sensing. 2021;180:174–90. doi: 10.1016/j.isprsjprs.2021.08.015

[46] Tucker CJ. Red and photographic infrared linear combinations for monitoring vegetation. Remote Sensing of Environment. 1979;8(2):127–50. doi: 10.1016/0034-4257(79)90013-0

[47] Ge G, Shi Z, Zhu Y, Yang X, Hao Y. Land use/cover classification in an arid desert-oasis mosaic landscape of China using remote sensed imagery: Performance assessment of four machine learning algorithms. Global Ecology and Conservation. 2020;22:e00971. doi: 10.1016/j.gecco.2020.e00971

[48] Qi J, Chehbouni A, Huete AR, Kerr YH, Sorooshian S. A modified soil adjusted vegetation index. Remote Sensing of Environment. 1994;48(2):119–26. doi: 10.1016/0034-4257(94)90134-1

[49] Shevtsova I, Heim B, Kruse S, Schröder J, Troeva EI, Pestryakova LA, et al. Strong shrub expansion in tundra-taiga, tree infilling in taiga and stable tundra in central Chukotka (north-eastern Siberia) between 2000 and 2017. Environmental Research Letters. 2020;15(8):85006. doi: 10.1088/1748-9326/ab9059

[50] Zha Y, Gao J, Ni S. Use of normalized difference built-up index in automatically mapping urban areas from TM imagery. International Journal of Remote Sensing. 2010;24(3):583–94. doi: 10.1080/01431160304987

[51] La Qu, Li M, Chen Z Zhi J. A Modified Self-adaptive Method for Mapping Annual 30-m Land Use/Land Cover Using Google Earth Engine: A Case Study of Yangtze River Delta. Chinese Geographical Science. 2021;31(5):782–94. doi: 10.1007/s11769-021-1226-4

[52] Breiman L. Random forests. Machine Learning. 2001;45(1):5–32. doi: 10.1023/a:1010933404324

[53] Congalton RG. A review of assessing the accuracy of classifications of remotely sensed data. Remote Sensing of Environment. 1991;37(1):35–46. doi: 10.1016/0034-4257(91)90048-b

[54] Fang P, Zhang X, Wei P, Wang Y, Zhang H, Liu F, et al. The Classification Performance and Mechanism of Machine Learning Algorithms in Winter Wheat Mapping Using Sentinel-2 10 m Resolution Imagery. Applied Sciences. 2020;10(15):5075. doi: 10.3390/app10155075

[55] Jiapaer G, Chen X, Bao A. A comparison of methods for estimating fractional vegetation cover in arid regions. Agricultural and Forest Meteorology. 2011;151(12):1698–710. doi: 10.1016/j.agrformet.2011.07.004

[56] Calvão T, Palmeirim JM. Mapping Mediterranean scrub with satellite imagery: biomass estimation and spectral behaviour. International Journal of Remote Sensing. 2010;25(16):3113–26. doi: 10.1080/01431160310001654978

[57] Dusseux P, Corpetti T, Hubert-Moy L, Corgne S. Combined Use of Multi-Temporal Optical and Radar Satellite Images for Grassland Monitoring. Remote Sensing. 2014;6(7):6163–82. doi: 10.3390/rs6076163

[58] Bartalev SA, Egorov VA, Loupian EA, Khvostikov SA. A new locally-adaptive classification method LAGMA for large-scale land cover mapping using remote-sensing data. Remote Sensing Letters. 2014;5(1):55–64. doi: 10.1080/2150704x.2013.870675

[59] Xuan F, Dong Y, Li J, Li X, Su W, Huang X, et al. Mapping crop type in Northeast China during 2013–2021 using automatic sampling and tile-based image classification. International Journal of Applied Earth Observation and Geoinformation. 2023;117. doi: 10.1016/j.jag.2022.103178

